# Exploration, novelty, surprise, and free energy minimization

**DOI:** 10.3389/fpsyg.2013.00710

**Published:** 2013-10-07

**Authors:** Philipp Schwartenbeck, Thomas FitzGerald, Raymond J. Dolan, Karl Friston

**Affiliations:** The Wellcome Trust Centre for Neuroimaging, Institute of Neurology, University College LondonLondon, UK

**Keywords:** active inference, exploration, exploitation, novelty, reinforcement learning, free energy

## Abstract

This paper reviews recent developments under the free energy principle that introduce a normative perspective on classical economic (utilitarian) decision-making based on (active) Bayesian inference. It has been suggested that the free energy principle precludes novelty and complexity, because it assumes that biological systems—like ourselves—try to minimize the long-term average of surprise to maintain their homeostasis. However, recent formulations show that minimizing surprise leads naturally to concepts such as exploration and novelty bonuses. In this approach, agents infer a policy that minimizes surprise by minimizing the difference (or relative entropy) between likely and desired outcomes, which involves both pursuing the goal-state that has the highest expected utility (often termed “exploitation”) and visiting a number of different goal-states (“exploration”). Crucially, the opportunity to visit new states increases the value of the current state. Casting decision-making problems within a variational framework, therefore, predicts that our behavior is governed by both the entropy and expected utility of future states. This dissolves any dialectic between minimizing surprise and exploration or novelty seeking.

## Introduction

The free energy principle is a theoretical formulation of biological systems and their behavior (Friston et al., 2006; Friston, 2009, 2010) that has attracted much current research interest (Brown and Friston, 2012; Adams et al., 2013a; Apps and Tsakiris, 2013; Joffily and Coricelli, 2013; Moran et al., 2013). Its underlying premise is that a biological system, in order to underwrite its existence and avoid the dispersion of its physical states, has to maintain its states within certain bounds and, therefore, maintain a homeostasis. Under ergodic assumptions this means that it has to minimize its long-term average surprise (i.e., Shannon entropy) over the states it visits. Surprise is an information theoretic quantity that can be approximated with variational free energy (Feynman, 1972; Hinton and van Camp, 1993). Every system that maintains itself conforms to the imperative of minimizing the surprise associated with the states it encounters. In the context of neuroscience, this implies that the brain becomes a model of the world in order to evaluate surprise in relation to model-based predictions (Friston, 2012). Practically, this means that it has to elaborate internal predictions about sensory input and update them based on prediction errors, a process that can be formulated as generalized Bayesian filtering or predictive coding in the brain (Friston, 2005). The notion of active inference translates predictive coding into an embodied context and argues that surprise can be minimized in two ways: either by optimizing internal predictions about the world (perception) or via acting on the world to change sensory samples so that they match internal predictions (action) (Brown et al., 2011).

The premise that every biological system—such as the brain—has to minimize variational free energy promises to provide a unified account of brain function and behavior and has proven useful for understanding neuroanatomy, neurophysiology (Feldman and Friston, 2010; Bastos et al., 2012; Brown and Friston, 2012; Adams et al., 2013b; Moran et al., 2013), and psychiatry (Edwards et al., 2012; Adams et al., 2013a). However, many recent discussions have deconstructed and critiqued the theory (Clark, 2013). In particular, a recurring criticism runs as follows: if our main objective is to minimize surprise over the states and outcomes we encounter, how can this explain complex human behavior such as novelty seeking, exploration, and, furthermore, higher level aspirations such as art, music, poetry, or humor? Should we not, in accordance with the principle, prefer living in a highly predictable and un-stimulating environment where we could minimize our long-term surprise? Shouldn't we be aversive to novel stimuli? As it stands, this seems highly implausible; novel stimuli are sometimes aversive, but often quite the opposite. The challenge here is to reconcile the fundamental imperative that underlies self-organized behavior with the fact that we avoid monotonous environments and actively explore in order to seek novel and stimulating inputs (Kakade and Dayan, 2002).

The free energy principle—under which our theoretical arguments are developed—is the quintessential normative theory for action and perception. It is normative in the sense that it provides a well-defined objective function (variational free energy) that is optimized both by action and perception. Having said this, the normative aspect of free energy minimization (and implicit active inference) is complemented by a neuronally plausible implementation scheme, in the form of predictive coding. We do not focus on the underlying imperatives for minimizing free energy (this has been fully addressed elsewhere). In this essay, we look specifically at the normative implications for behavior in the context of classical (economic) decision-making problems. Our normative account argues that optimal decisions minimize the relative entropy between likely and desired outcomes. This means that—in some contexts—agents are compelled to seek novel states, whereas in other contexts they maximize expected utility. We hope to show that explorative behavior is not just in accordance with the principle of free energy minimization but is in fact mandated when minimizing surprise (or maximizing model-evidence) in the context of decision-making behavior. In brief, we argue that when a policy (i.e., an action selection rule that entails a sequence of actions) is selected—in a way that includes uncertainty about outcomes—there is necessarily an exploratory drive that accompanies the classical maximization of expected utility.

## Boredom and novelty seeking under the free energy principle

When addressing this issue, one has to appreciate an important but subtle difference between two questions: one being why the imperative to minimize surprise does not predict that we seek out an impoverished or senseless environment; the other being how the free energy principle motivates the active exploration of new states. The former question is associated with the “dark room problem,” which has been dealt with previously (Friston et al., 2009, 2012). The “dark room problem,” however, does not refer to a real “problem” but merely a misapprehension about what is meant by “surprise”: it can be easily resolved by appreciating the difference between minimizing the long-term surprise over states (i.e., the Shannon entropy) *H*[*S*] *per se* compared to minimizing the long-term surprise given a specific (generative) model: *H*[*S*|*m*]. This means that agents are equipped with prior beliefs—which can be innate and acquired by natural selection such as an aversion to hypoglycaemia or dehydration or shaped by learning according to experience (Friston, 2011, 2013)—that define what an agent regards as surprising. Put simply, (most) agents would find it highly surprising to be incarcerated in a dark room and would thus generally try to avoid that state of affairs. More formally, there is a fundamental difference between the intuitive meaning of “surprise” in terms of unpredictable sensory input and surprise (in information theoretic terms) under a particular model of the world. Finding ourselves in a dark room (and being subject to a surprising sense of starvation and sensory deprivation) is a highly surprising state, even though it represents an environment with maximally predictable sensory input.

It is reassuring that the free energy principle does not compel us to seek an empty room, turn off the light and wait there until we die. However, what does it have to say concerning autonomous, purposeful behavior and why we actively aspire (to a certain extent) to novel, complex states? Why do we enjoy going to exhibitions and seeing our favorite piece of art—or learning about new artists—when our main objective is to restrict our existence to a limited number of (attractor) states to maintain a homeostasis?

This question is addressed in a recent application of the free energy framework, which casts complex, purposeful decision-making as active inference (Friston et al., 2013). The basic assumption that action minimizes surprise by selective sampling of sensory input (to match internal predictions) is applied to fictive states in the future. Put simply, this means that an agent's prior beliefs include the notion that it will act to minimize surprise. By analogy to perceptual inference—where agents are equipped with a generative model mapping from hidden causes to sensory consequences—the agent's generative model includes hidden (future) states and actions that the agent might perform (and their consequences). This implies that the agent has to represent itself in future states performing specific actions. In other words, it necessarily implies a model with a sense of agency.

Based on its generative model, the agent has to infer policies in order to minimize surprise about future outcomes. Beliefs about the (optimal) policies it will find itself pursuing is based on their value, which can be expressed in the following probabilistic terms:

$$Q\left(\pi |s_{t}\right)=-D_{KL}\left[P\left(s_{T}|s_{t},\pi \right)\|P\left(s_{T}|m\right)\right]$$

Equation 1 formalizes the intuitive notion that valuable policies minimize the difference between likely and desired outcomes by bringing the former as close as possible to the latter. The left side of the equation refers to the value *Q* of a given policy π from a specific state at time *t* ϵ *T*. The right side of the equation defines this value as the (negative) difference or relative entropy (Kullback-Leibler Divergence) between two probability distributions: *P*(*s*_*T*_|*s*_*t*_, π) refers to a probability distribution over outcome states, given a specific policy and a current state, whereas *P*(*s*_*T*_|*m*) refers to a probability distribution over outcomes based solely on the prior beliefs or intentional goals of the agent. The former distribution refers to the empirical prior over outcomes given a specific sequence of actions the agent might perform, whereas the latter refers to priors that represent which goal state the agent believes it will (desires to) attain. These goal priors are fixed and do not depend on sensory input: they represent a belief concerning states the agent will end up in. Desired goal states will be accorded a high probability (log-likelihood) of being encountered, resulting in a low surprise when this state is indeed visited. Undesirable states, by contrast, will be assigned with a low prior probability and therefore become highly surprising.

Crucially, casting decision-making as KL control (or equivalently surprise or relative-entropy minimization) subsumes classical notions of reward and utility that are central to fields such as behavioral economics and reinforcement learning, since the value of a state becomes simply a function of how surprising it is: visiting unsurprising states is associated with a high reward in classical reinforcement and utilitarian schemes, whereas surprising future states have low reward or high cost. In the framework of active inference, therefore, agents do not try to maximize reward but minimize surprise (about future states). Similar accounts following KL control have been proposed earlier (Solway and Botvinick, 2012; Huang and Rao, 2013), but in the context of prior beliefs over the magnitude of rewards in future states. Here, valuable policies minimize the Kullback-Leibler divergence (relative entropy) between the distribution of likely outcomes and the distribution of desired outcomes represented as belief about attaining them (which we assume to be fixed and defined a priori).

To see why this scheme mandates both exploitation (value maximization) and exploration (visiting novel states), one can rewrite this KL-Divergence term as:

$$\begin{array}{l} -D_{KL}\left[P\left(s_{T}|s_{t},\pi \right)\|P\left(s_{T}|m\right)\right]=\sum_{s_{T}}P\left(s_{T}|s_{t},\text{​}\pi \right)\ln\frac{P\left(s_{T}|m\right)}{P(s_{T}|s_{t},\text{​}\pi )}\\ \;=\sum_{s_{T}}(-P\left(s_{T}|s_{t},\text{​}\pi \right)\cdot \ln P\left(s_{T}|s_{t},\text{​}\pi \right)+\;P\left(s_{T}|s_{t},\text{​}\pi \right)\cdot \ln P\left(s_{T}|m\right))\\ \;=-\sum_{s_{T}}\left(P\left(s_{T}|s_{t},\text{​}\pi \right)\cdot \ln P\left(s_{T}|s_{t},\text{​}\pi \right)\right)+\sum_{s_{T}}\left(P\left(s_{T}|s_{t},\text{​}\pi \right)\cdot \ln P\left(s_{T}|m\right)\right)\\ \;=H\text{​}\left[P(s_{T}|s_{t},\text{​}\pi )\right]+\sum_{s_{T}}P\left(s_{T}|s_{t},\text{​}\pi \right)\cdot u\left(s_{T}|m\right) \end{array}$$

**Figure 1:**
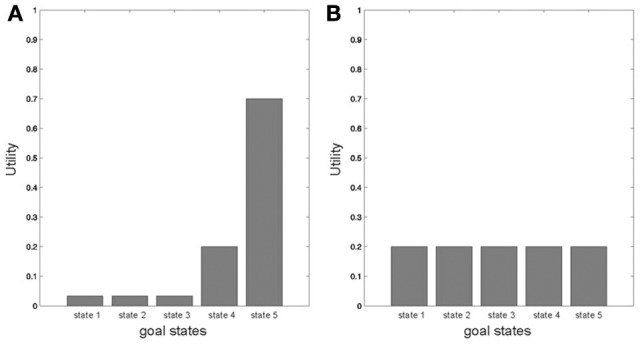
**Illustrating two different contexts in which a valuable policy is defined according to the likelihood of visiting a goal state with highest utility (A–because one goal state is clearly favored by the agent) or according to the likelihood of visiting many different states (B–because no goal state is favored), depending on the different representations of utilities of goal states in an agent's prior beliefs**.

This decomposition of the value of a policy is important as it speaks to two different ways of maximizing the value of a selected policy: the first term is the entropy over goal-states, which reflects the number of different outcomes the agent is likely to experience under a specific policy, whereas the second term represents the expected utility over outcomes that depends on an agent's priors *u* (*s*_*T*_|*m*) = ln *P*(*s*_*T*_|*m*), which constitute an agent's goals and the (beliefs about) utility of final states. This term increases the value of a policy that secures the outcome with highest expected utility. The relative contribution of these two to the value of a policy depends on the current state and the precision with which prior beliefs about goals are held, as illustrated in Figure 1. When the utilities of outcomes differ (and are well-defined), a policy that makes visiting the outcome with highest utility (and only this one) most likely will be the most valuable. When outcomes have the same or similar utilities, on the other hand, policies cannot be differentiated according to expected utility. In this case, policies will be valuable if they maximize the entropy over outcome states in accordance with the maximum entropy principle (Jaynes, 1957), which means the agent will try to visit all states with equal probability.

The notion behind this decomposition goes beyond stating that agents maximize entropy if the utilities of outcomes are the same and maximize expected utility if they differ, but rather implies that all our decisions are influenced by entropy and expected utility—with a context-sensitive weighting of those two. This decomposition may account for numerous instances of every-day choice behavior, such as why we appreciate variation over outcomes much more when we buy a chocolate bar as opposed to a car: when the differences in the expected utilities of outcomes become less differentiable, agents will try to visit several states and not just the state that has highest utility.

This distinction is interesting because it maps to various other accounts of complex decision-making and planning. Most importantly, this distinction resembles exploration-exploitation (Sutton and Barto, 1998; Cohen et al., 2007), which is prominent in reinforcement learning paradigms. Here, choosing a policy that maximizes expected utility corresponds to exploitation, whereas maximizing entropy over outcomes corresponds to exploration. An important difference is, however, that exploration is often equated with random or stochastic behavior in reinforcement learning schemes (but see Thrun, 1992), whereas in our framework, maximizing entropy over outcome states is a goal-driven, purposeful process—with the aim of accessing allowable states. Furthermore, this distinction neatly reflects the differentiation between intrinsic and extrinsic reward (Schmidhuber, 1991, 2009; Luciw et al., 2013), where extrinsic reward refers to externally administered reinforcement—corresponding to maximizing expected utility—and intrinsic reward is associated with maximizing entropy over outcomes. Maximizing intrinsic reward is usually associated with seeking new experiences in order to increase context-sensitive learning—which is reflected as increasing model-evidence or minimizing surprise in the active inference framework.

The formal difference between classical (utilitarian) formulations of valuable behavior and those that are deemed valuable under active inference can be reduced to a simple distinction: in classical schemes, policies are chosen to maximize expected utility, whereas in active inference they are chosen to minimize the probabilistic divergence between controlled outcomes and a probability distribution that is defined in terms of utility. This difference induces an entropy or exploration term that would require some *ad*-*hoc* augmentation of classical utility functions. However, there is something more fundamental about the different approaches. Recall from above that policies are inferred during active inference. In other words, the agent has to infer which policy it is most likely to pursue and then selects that policy. Because this formulation converts an optimal control or reinforcement learning problem into an inference problem, beliefs about optimal policies can themselves be optimized in terms of their precision or confidence. This precision corresponds to the temperature or sensitivity parameter in classical models that appeal to softmax choice rules. This is important because precision can be optimized in a Bayes optimal sense during active inference and ceases to be an *ad-hoc* or descriptive parameter of choice behavior. In Friston et al. (2013) we show that the updating of precision has many of the hallmarks of dopamine discharges.

## Conclusion

The aim of this paper was to explain exploration and novelty seeking under the free energy principle. The formalism presented here is part of a general framework of decision making as active inference and will be discussed in more detail elsewhere (Friston et al., 2013). This theoretical piece serves to underlie the basic issues and potential ways forward. It will be complemented by a series of more technical papers (based on simulations, empirical studies of choice behavior and functional neuroimaging) that provide specific examples and operationalize the ideas discussed in the current overview. We have shown that concepts like intrinsic and extrinsic reward—or exploration and exploitation—emerge naturally from casting decision-making under the normative assumption that agents minimize the relative entropy (KL-divergence) between likely and desired outcomes. Valuable policies will maximize expected utility or entropy over outcomes (or both), where the relative weight of these two mechanisms is context specific and depends upon prior beliefs.

We therefore resolve an apparent paradox concerning the incompatibility of minimizing surprise and the exploration of novel states, which constitute an essential aspect of human and animal behavior. Indeed, under certain circumstances, surprise can be minimized (i.e., model evidence can be maximized) if an agent selects a policy that increases the likelihood of visiting new and informative states. The concept of surprise minimization, therefore, by no means precludes agents from active exploration or appreciating novelty but rather explicitly predicts that this is an important factor in guiding our behavior. The most straightforward application of the formalism presented here clearly lies in economic decision-making tasks. Our formalism is certainly not sufficient–in the given form–to explain all aspects of higher level activities, such as the appreciation of fine arts. Maximizing intrinsic reward and visiting new and informative states to maximize model evidence (i.e., improve our model of the world) may, however, lay the foundation for future developments along these lines. Furthermore, empirical research is currently investigating the relative influence of entropy and expected utility maximization on behavior and their association with neuronal activation—which may well be related to specific personality traits such as sensation seeking. We look forward to reporting these results in the not too distant future.

### Conflict of interest statement

The authors declare that the research was conducted in the absence of any commercial or financial relationships that could be construed as a potential conflict of interest.
